# Supplementary material for: The Influence of Exogenous CdS Nanoparticles on the Growth and Carbon Assimilation Efficiency of Escherichia coli
Source: Biology (Basel). 2024 Oct 21;13(10):847. doi: 10.3390/biology13100847 (PMC11505546; doi:10.3390/biology13100847)
Supplement: Supplementary file 1 [file biology-13-00847-s001.zip › biology-3204904-supplementary.pdf]

Inhibitory and growth-promoting effects of CdS NPs on microorganisms: ecological implications

Authors:

Kuo Yang[1,2,**], Yue Yang[1,2,**], Jie Wang[1,2,**], Xiaomeng Huang[1,2], Daizong Cui[1,2,*], Min Zhao[1,2,*]

Affiliations:

[1]College of Life Science, Northeast Forestry University, Harbin, 150000, China.

[2]Key Laboratory for Enzyme and Enzyme-like Material Engineering of Heilongjiang, Harbin, 150000, China

[*]Corresponding author. Daizong Cui: siyu19831114@163.com; Min Zhao: 82191513@163.com.

**These authors contributed equally: Kuo Yang, Yue Yang, Jie Wang.

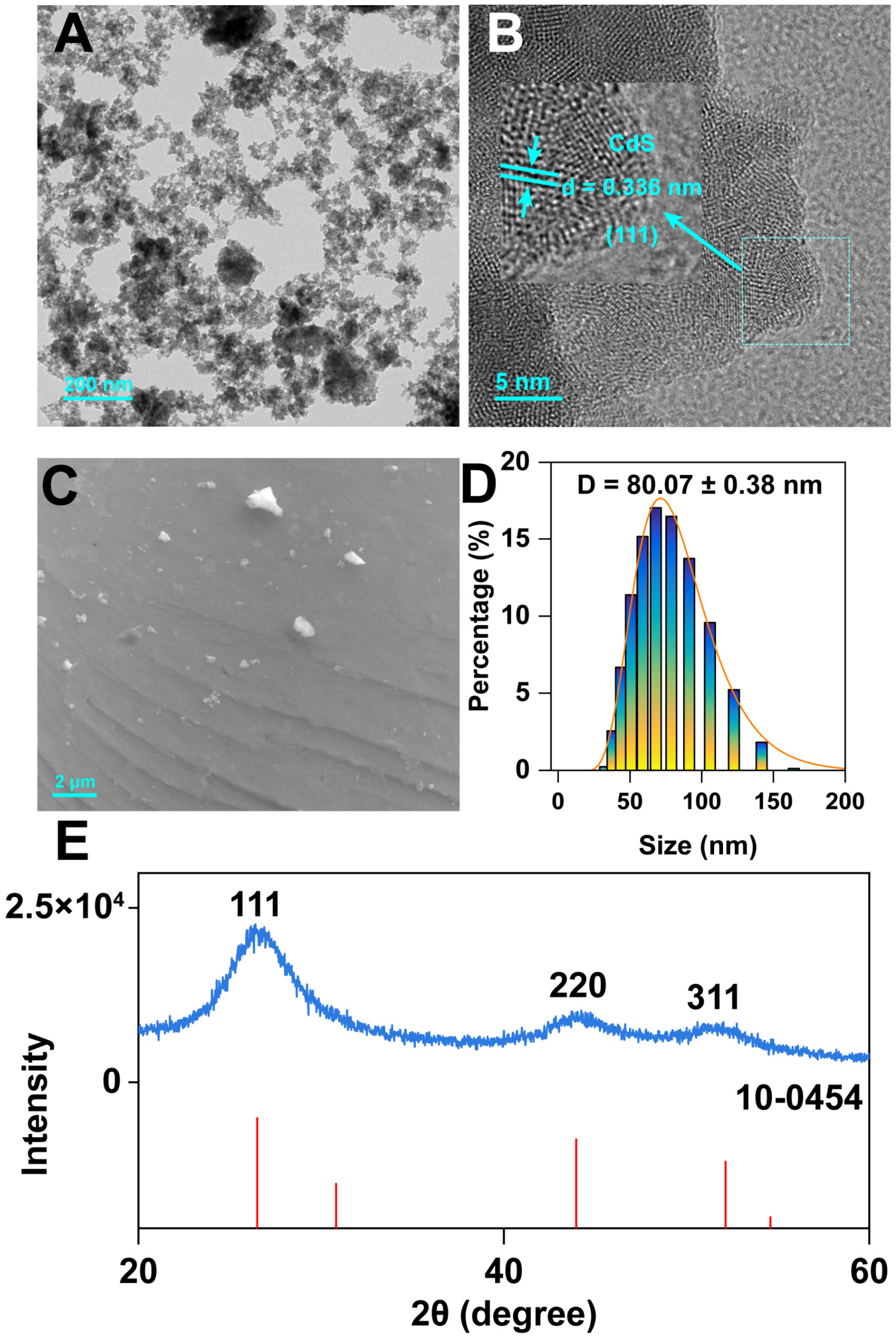

Fig. S1 TEM image of (A) CdS NPs, (B) HRTEM image of CdS NPs, (C) SEM image of CdS NPs at 2 μm and (D) the particles size distribution. (E) XRD patterns of CdS NPs.

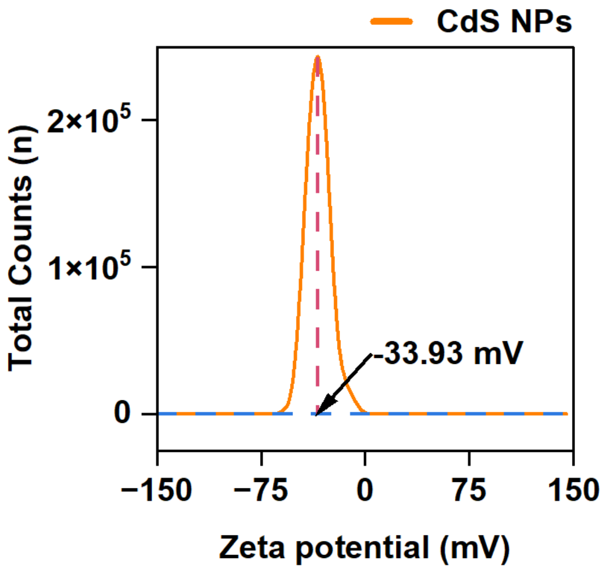

Fig. S2 Zeta potential analysis of CdS NPs.

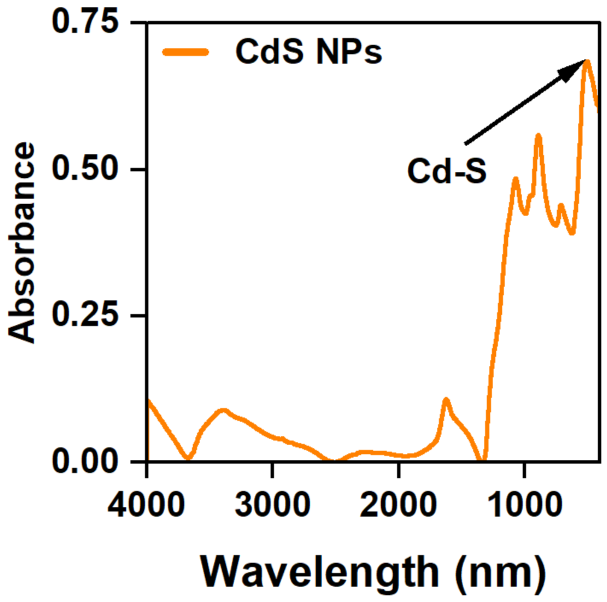

Fig. S3 FTIR analysis of CdS NPs.

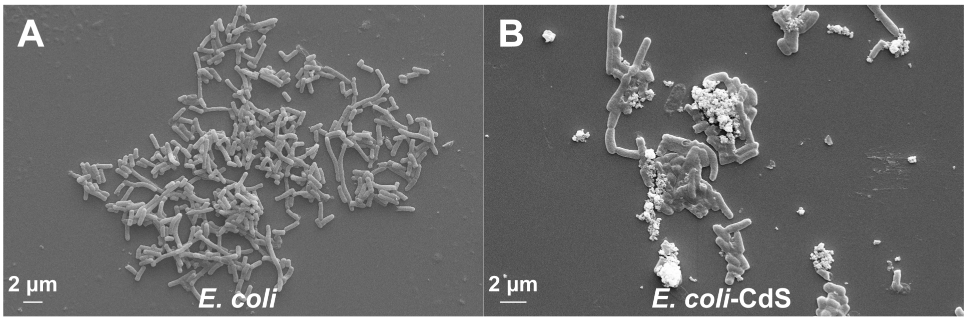

Fig. S4 SEM images of (A) *E. coli* and (B) *E. coli*-CdS.

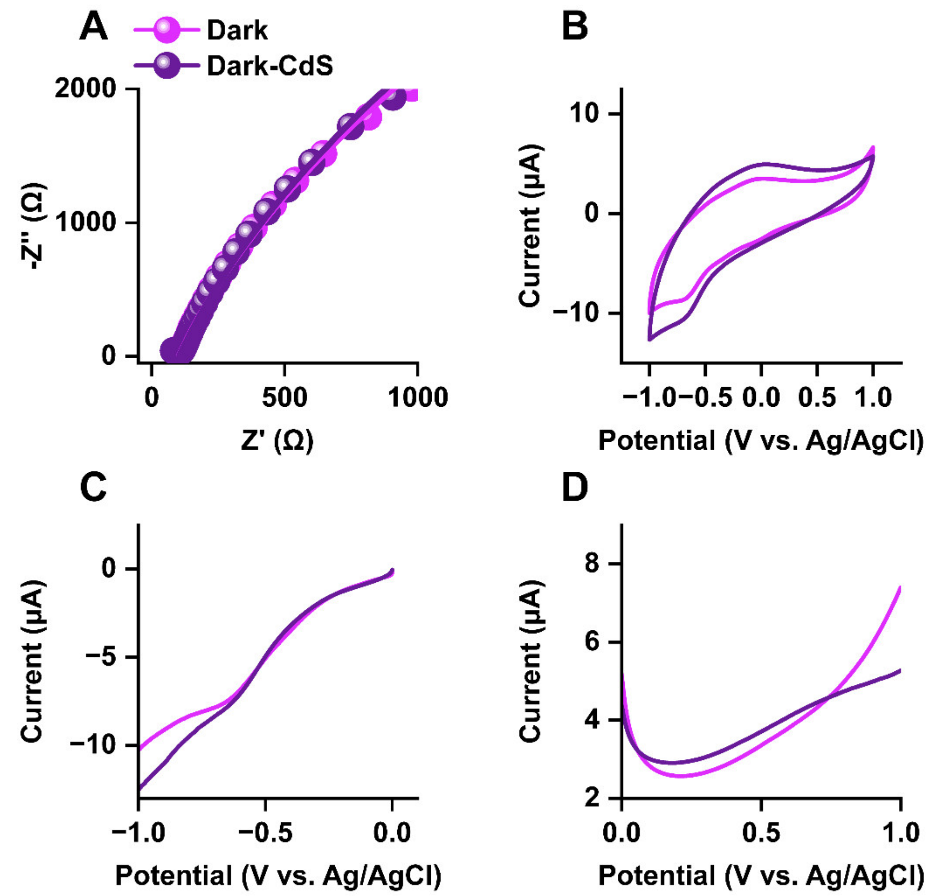

Fig. S5 Nyquist plot of (A) the samples in dark environment and fitted data (solid line). (B) Cyclic voltammogram of *E. coli* cultured under dark condition with or without CdS NPs. (C) Cathodic LSV curves on *E. coli* with or without CdS NPs in dark groups, (D) anodic LSV curves on *E. coli* with or without CdS NPs in dark groups.

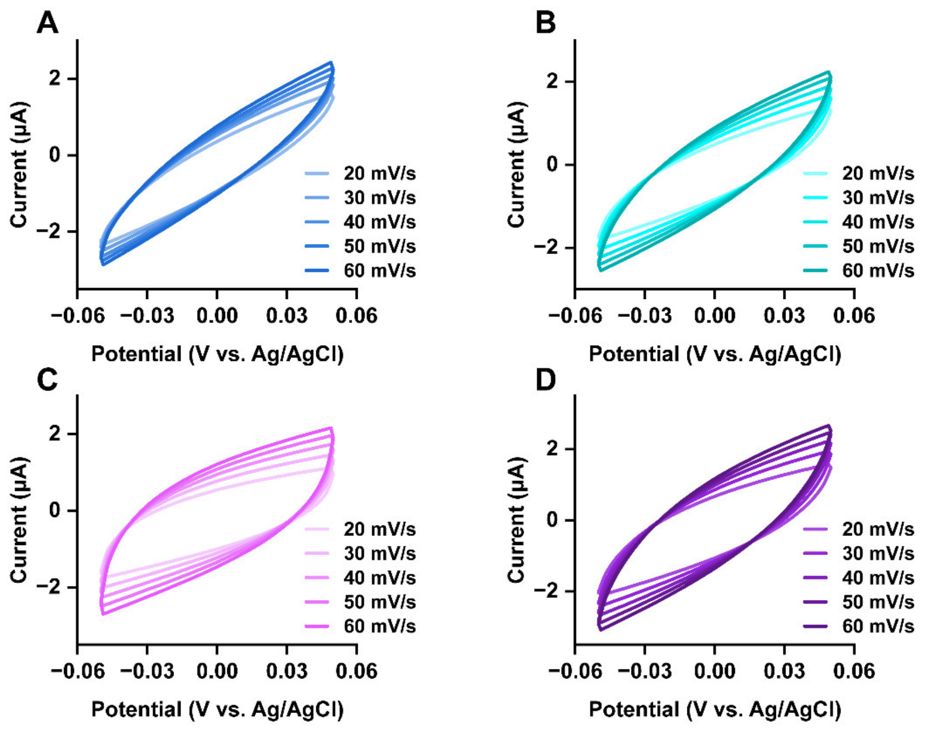

Fig. S6 Cyclic voltammogram of *E. coli* cultured (A) with or (B) without CdS NPs in light or (C-D) dark condition under scan rate at 20, 30, 40, 50, 60 mV/s.

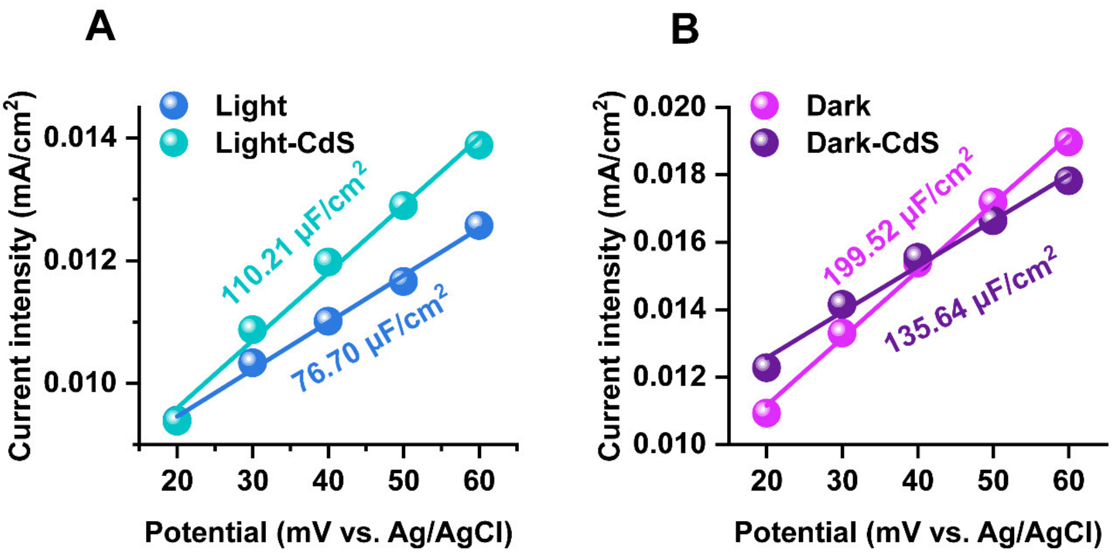

Fig. S7 The ECSA values of (A) *E. coli* with or without CdS NPs in light or (B) dark illumination under different treatments were evaluated from the slope of the fitting function of capacitive current.

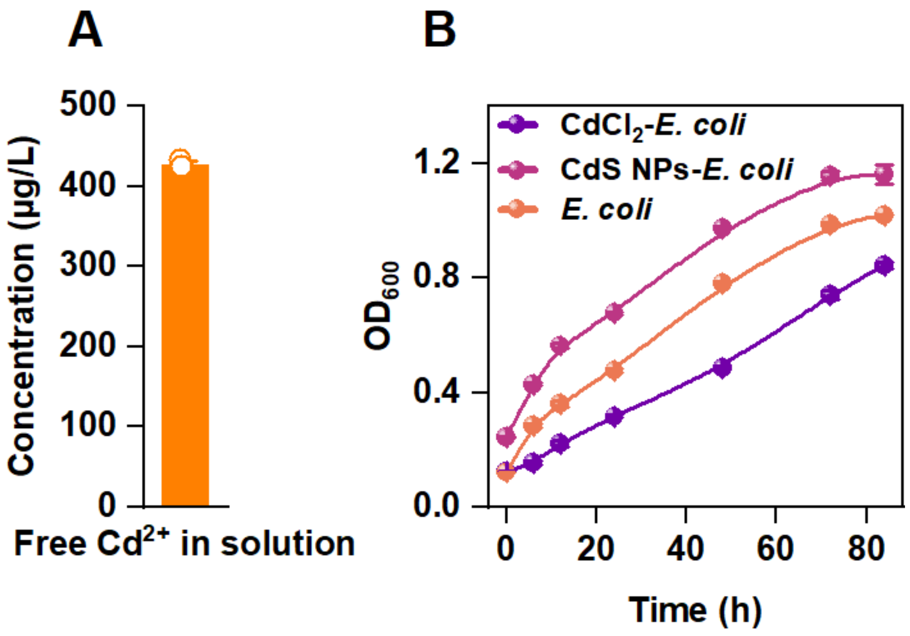

Fig. S8 (A) The concentration of Cd^{2+} released in the culture medium from CdS-*E. coli*. (B) The bacterial growth curves of different treatment groups.

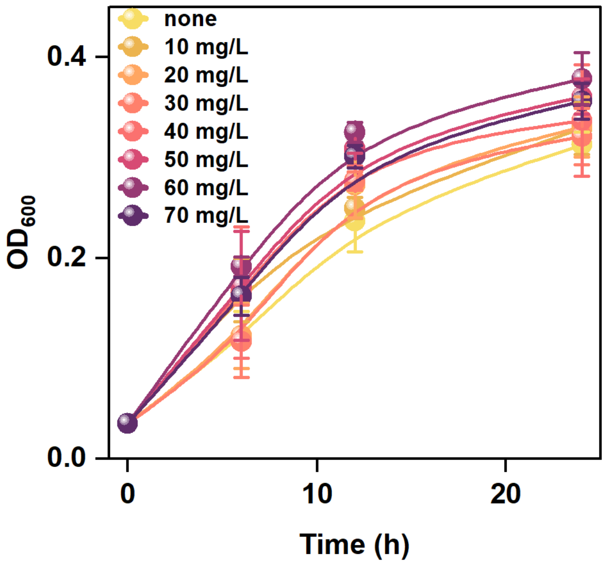

Fig. S9 Effect of different concentrations of CdS on *E. coli* growth.

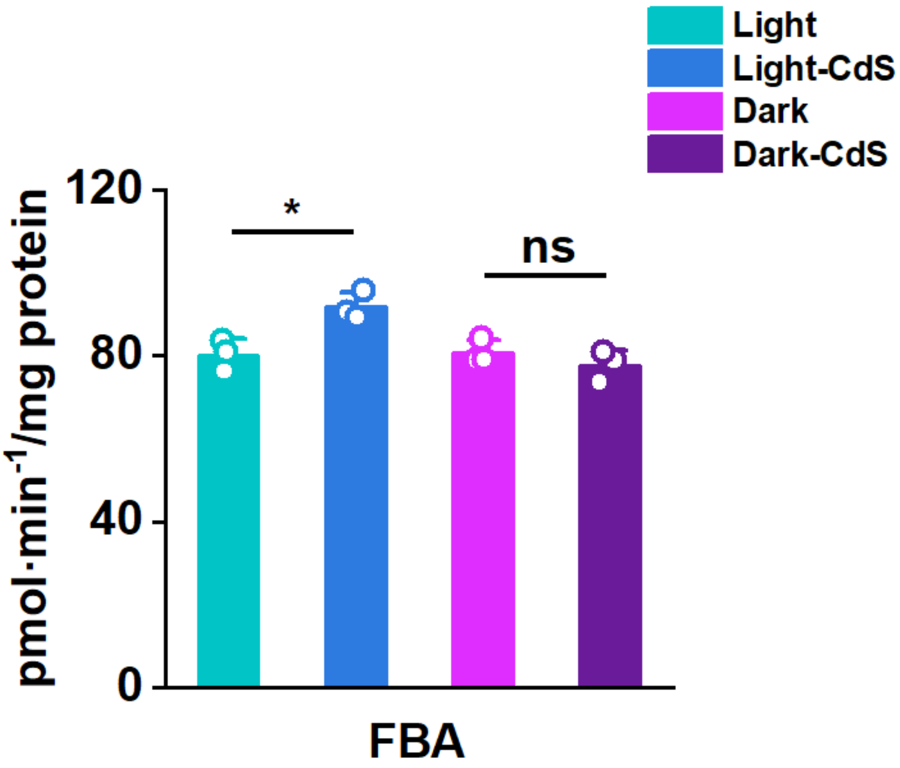

Fig. S10 Differences in the activity units of Fructose-1,6-bisphosphate aldolase in carbon metabolism by different treatment groups.

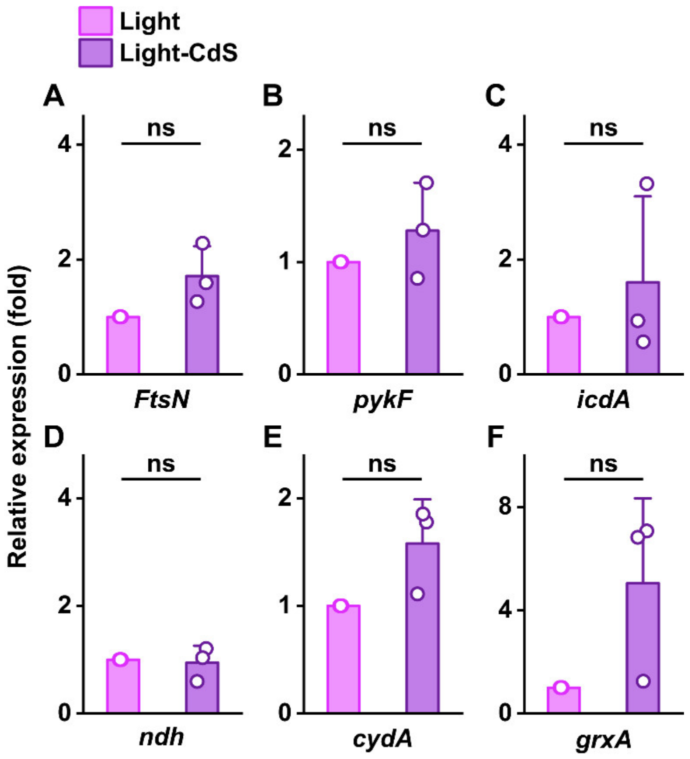

Fig. S11 The relative expression levels of (A) *FtsZ*, (B) *pykF*, (C) *icdA*, (D) *ndh*, (E) *cydA*, (F) *grxA* in *E. coli* under dark environment. *ns* > 0.05, *n* = 3 for each group.

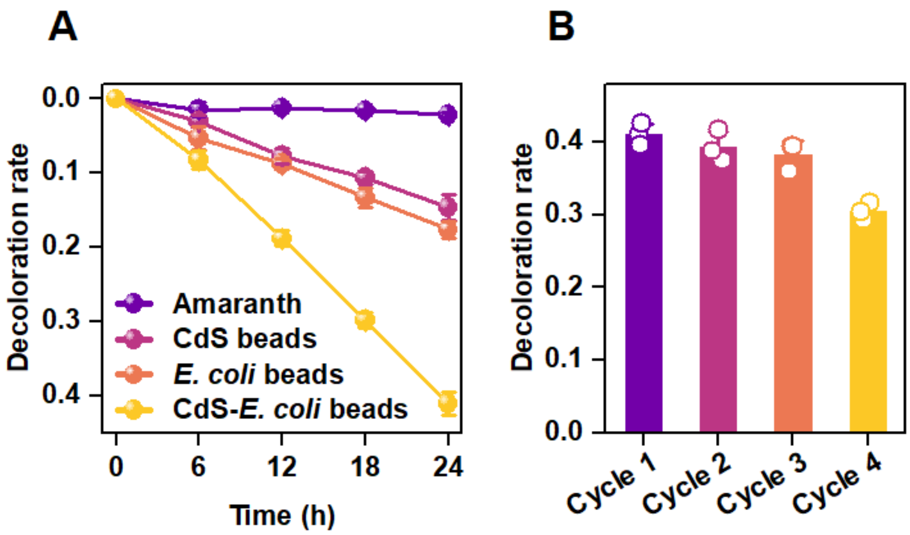

Fig. S12 (A) Dye decolorization in different treatment groups and (B) the cyclic decolorization performance of CdS-*E. coli* encapsulated beads on amaranth dye.

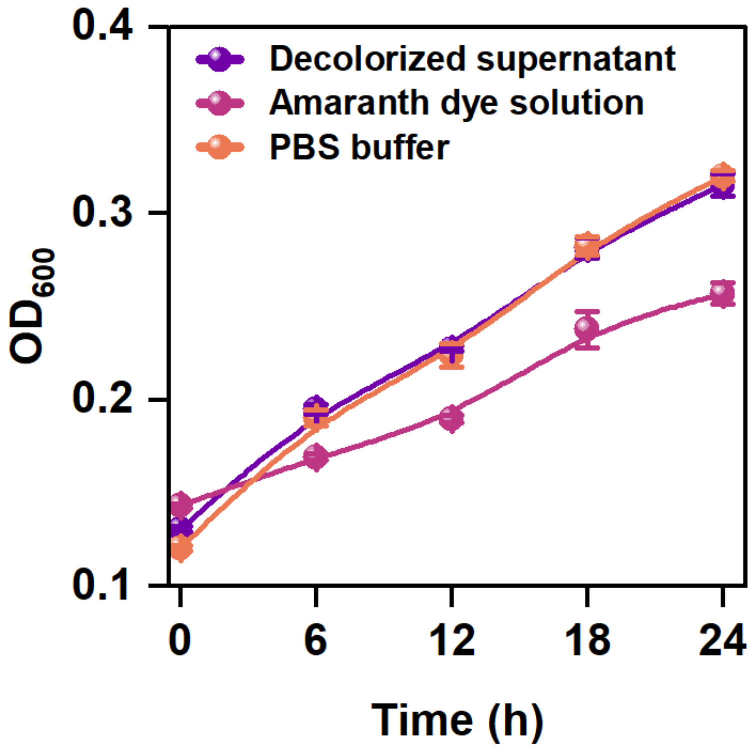

Fig. S13 Biological safety assessment of CdS-*E. coli* encapsulated beads.

Table. S1 The primer sequences of the target genes for qRT-PCR analysis

Target genes	Primer sequences
FtsN	Forward Primer: 5'-GTTTTCACTTCACCACCGGC-3'
	Reverse Primer: 5'-CGGAAACGGACTACCACCAA-3'
pykF	Forward Primer: 5'-CCTGGGCGAAAACAAAGGTG-3'
	Reverse Primer: 5'-AGTCTACGCCTTGTTCGCAA-3'
icdA	Forward Primer: 5'-AGTTCACCGAAGGAGCGTTT-3'
	Reverse Primer: 5'-TCTTTGCCAGTGTTCGGGTT-3'
ndh	Forward Primer: 5'-TCGCCTATGACACCCTGGTA-3'
	Reverse Primer: 5'-CCCAGGTTGGCGGAGTATTT-3'
cydA	Forward Primer: 5'-CTGTCCGCACTGTGGATTCT-3'
	Reverse Primer: 5'-ACCTGAGCAACCGGGTTAAG-3'
grxA	Forward Primer: 5'-CGTGCGGAAGGGATCACTAA-3'
	Reverse Primer: 5'-TCGACAAAAATCTGCGGCAC-3'
16s rDNA	Forward Primer: 5'-TGGTCTGAGAGGATGACCAG-3'
	Reverse Primer: 5'-TGCTTCTTCTGCGGGTAACG-3'

Table. S2 Capacitance of each culture tested in CV

	Light	Light-CdS	Dark	Dark-CdS
Capacitance/μF	12.92	17.20	10.07	12.69
